# Brachial Artery Flow-mediated Dilation Following Exercise with Augmented Oscillatory and Retrograde Shear Rate

**DOI:** 10.1186/1476-7120-10-34

**Published:** 2012-08-11

**Authors:** Blair D Johnson, Kieren J Mather, Sean C Newcomer, Timothy D Mickleborough, Janet P Wallace

**Affiliations:** 1 Department of Kinesiology, Indiana University, Bloomington, IN, USA; 2 Department of Medicine, Indiana University, Indianapolis, IN, USA; 3 Department of Health and Kinesiology, Purdue University, West Lafayette, IN, USA; 4 Department of Anesthesiology, 200 First Street SW, SMH, Joseph 4-184, Rochester, MN, 55905, USA

**Keywords:** Antegrade, Antioxidant, Oxidative stress, Supine cycle ergometer

## Abstract

**Background:**

Acute doses of elevated retrograde shear rate (SR) appear to be detrimental to endothelial function in resting humans. However, retrograde shear increases during moderate intensity exercise which also enhances post-exercise endothelial function. Since SR patterns differ with the modality of exercise, it is important to determine if augmented retrograde SR during exercise influences post-exercise endothelial function. This study tested the hypothesis that (1) increased doses of retrograde SR in the brachial artery during lower body supine cycle ergometer exercise would attenuate post-exercise flow-mediated dilation (FMD) in a dose-dependent manner, and (2) antioxidant vitamin C supplementation would prevent the attenuated post-exercise FMD response.

**Methods:**

Twelve men participated in four randomized exercise sessions (90 W for 20 minutes) on separate days. During three of the sessions, one arm was subjected to increased oscillatory and retrograde SR using three different forearm cuff pressures (20, 40, 60 mmHg) (contralateral arm served as the control) and subjects ingested placebo capsules prior to exercise. A fourth session with 60 mmHg cuff pressure was performed with 1 g of vitamin C ingested prior to the session.

**Results:**

Post-exercise FMD following the placebo conditions were lower in the cuffed arm versus the control arm (arm main effect: *P* < 0.05) and without differences between cuff pressures (20 mmHg: 5.7 ± 2.2%; 40 mmHg: 4.7 ± 1.3%; 60 mmHg: 5.4 ± 2.4%) (*P* > 0.05). Following vitamin C treatment, post-exercise FMD in the cuffed and control arm increased from baseline (*P* < 0.05) but were not different (control: 7.1 ± 3.5% vs. cuffed: 6.6 ± 3.3%) (*P* > 0.05).

**Conclusions:**

These results indicate that augmented oscillatory and retrograde SR in non-working limbs during lower body exercise attenuates post-exercise FMD without an evident dose–response in the range of cuff pressures evaluated. Vitamin C supplementation prevented the attenuation of FMD following exercise with augmented oscillatory and retrograde SR suggesting that oxidative stress contributes to the adverse effects of oscillatory and retrograde shear during exercise on FMD.

## Background

Improvements in recognized traditional and novel risk factors account for approximately 59% of the benefits associated with exercise which leaves a large portion of the exercise-induced reduction of disease risk unexplained [1]. Exercise provides direct beneficial effects to the vasculature which may contribute to the unexplained cardiovascular disease risk reduction [2]. Data obtained from animals and humans demonstrate that exercise produces an increased superoxide dismutase expression/activity [3], improved endothelial nitric oxide synthase (eNOS) expression and phosphorylation [4], enhanced acetylcholine-induced vasomotor function [5], and a reduction in pro-oxidant enzymes [3,6]. These beneficial effects on the endothelium may be a result of exercise-induced shear stress, which has been postulated to directly contribute to the improved function of the endothelium following exercise [2,7].

Oscillatory and retrograde shear stress appear to adversely influence endothelial cells *in vitro* and these effects have been studied extensively. Retrograde and oscillatory shear stress profiles increase mRNA of adhesion molecules [8,9], endothelin-1 [10], monocyte chemotactic protein 1 [9,11], gp91phox [11], Nox4 and reactive oxygen species [11,12] and decrease eNOS mRNA [11]. These shear patterns also stimulate mitochondrial production of reactive oxygen species [13]. Superoxide anions generated through these pathways also rapidly react with NO to form peroxynitrite [14], which uncouples eNOS by oxidizing tetrahydrobiopterin (BH_4_). Uncoupled eNOS further increases superoxide anion production leading to a vicious cycle of reactive oxygen species production [15] and decreased NO bioavailability [15]. Oxidative stress in endothelial cells is reduced and eNOS function is preserved when the antioxidant vitamin C is administered during oxidative challenges [16,17]; therefore it is reasonable to assume that reducing oxidative stress generated by oscillatory and retrograde shear by antioxidant supplementation may preserve NO bioavailability.

In humans, acute periods of augmented oscillatory and retrograde shear accompanied with a reduction of mean shear at rest have been observed to impair endothelial function in a retrograde shear dose-dependent manner [18]. Shear profiles in non-working limbs during exercise appear to vary with the mode of exercise [19], aerobic exercise intensity [20], exercise duration [21], and these effects differ across vessels [22]. Both antegrade and retrograde shear in the brachial artery are augmented during lower body exercise [20]. Despite the increased retrograde shear during exercise, endothelial function following moderate intensity aerobic exercise is increased [23,24]. Recently, it has been hypothesized that elevated antegrade shear during exercise counteracts the negative effects of retrograde shear allowing for an improvement of endothelial function following exercise [2]. Therefore, evaluating how various shear profiles during exercise impact post-exercise endothelial function is important given the differences in shear profiles during various exercise modalities and intensities. In this context, we tested the hypothesis that post-exercise brachial artery flow-mediated dilation (FMD) would be attenuated in a dose-dependent manner by varying the amount of retrograde shear in the brachial artery during lower body supine exercise. Based on *in vitro* findings [11,12,14,15], we also sought to explore the possible role of oxidative stress in mediating FMD following exercise with increased oscillatory and retrograde shear profiles. Accordingly, we hypothesized that vitamin C supplementation would prevent the attenuation of brachial artery FMD following elevated brachial artery oscillatory and retrograde shear during lower body supine exercise.

## Methods

### Subjects

Twelve healthy men volunteered to participate in three screening visits followed by four exercise sessions. During the first screening visit subjects gave written informed consent, completed a medical history/health habits questionnaire followed by the assessment of height, weight, and resting blood pressure. During the second screening visit, an additional blood pressure measurement was made. The third screening visit consisted of an appointment with the University Health Center where a fasting venous blood sample was collected. All subjects self-reported to participate in aerobic exercise, were non-smokers, and were not taking any vaso-active medications, anti-hypertension medications, lipid lowering drugs, or anti-diabetic medications, and were stratified as low cardiovascular disease risk by the American College of Sports Medicine Risk Stratification criteria [25]. Subjects also discontinued any antioxidant supplementation three days prior to the first exercise session and were asked to maintain their normal dietary habits throughout the remainder of the study. All procedures were approved by the University Committee for the Protection of Human Subjects.

### Experimental design

After confirming eligibility, subjects completed 4 exercise sessions separated by 1 to 7 days. Subjects were asked to refrain from exercise, alcohol, tobacco products, and food/drinks that contain caffeine for at least 8 h and reported to the laboratory at the same time of day for all sessions (between 0700 and 0930) following an overnight fast (> 6 h). Sessions were performed in a randomized double-blind, placebo controlled, cross-over design. One arm was randomly chosen to be subjected to an increased oscillatory and retrograde shear rate (SR) in the brachial artery while the contralateral arm served as the control during lower body cycle ergometer exercise. The same arm assignments were used for all four 20 minute exercise sessions for each subject. Upon arrival to the laboratory, subjects selected a container at random which enclosed information pertaining to the session (i.e. amount of forearm cuff pressure and which arm would be cuffed was revealed to the investigator) and two 500 mg sucrose placebo capsules or two 500 mg vitamin C capsules. The capsules were identical in color, weight, and size and were coded so the content of the capsules was unidentified until after data analysis was complete. The subjects ingested one placebo capsule 90 minutes and one placebo capsule 120 minutes (total: 1000 mg sucrose) prior to baseline testing for three exercise sessions (20 mm Hg, 40 mm Hg, and 60 mm Hg forearm cuff pressure). An additional exercise session with 60 mm Hg forearm cuff compression was included in the randomization, but subjects consumed a vitamin C capsule at 90 min and a vitamin C capsule 120 minutes (total: 1000 mg vitamin C (NOW® Foods, Bloomingdale, IL)) prior to baseline testing. Brachial artery FMD was used as an index of endothelial function and was measured in both arms prior to and immediately following the exercise sessions.

### Exercise sessions

Each subject completed 4 supine lower body cycle ergometer exercise sessions. A 5 × 84 cm automatic blood pressure cuff (E-20 rapid cuff inflator; D.E. Hokanson, Bellevue, WA, USA) was placed around both forearms immediately distal to the antecubital space and the arms were extended laterally and secured in custom-designed arm immobilizers. After baseline FMD was assessed in both arms, one cuff was randomly selected and inflated to a randomly chosen pressure of 20 mm Hg, 40 mm Hg, or 60 mm Hg (all cuff pressures were below resting diastolic blood pressure for all subjects) during 20 minutes of lower body supine cycling exercise at 90 W. Brachial artery antegrade and retrograde blood velocities and arterial diameters were measured during the exercise sessions every 5 minutes for the calculation of SR using a high-resolution ultrasound with concurrent Doppler flow measurements. Comparable interventions have been used to alter SR profiles in the brachial artery at rest and during exercise which attenuate post-intervention brachial artery FMD [18,23]. The cuff pressure during exercise was released immediately following the 20 minute exercise session. A three lead EKG was used to measure heart rate every 5 minutes throughout the exercise sessions.

### Brachial artery flow-mediated dilation

Brachial artery FMD was assessed as an index of endothelial function using current guidelines [26]. A 20 minute acclimatization phase was used prior to baseline FMD assessments in order to obtain a steady hemodynamic state. The first brachial artery assessed was randomly chosen. Images of the brachial artery were acquired longitudinally 2–10 cm above the antecubital space by a 2D high resolution ultrasound system (Terason T3000, Teratech Corp., Burlington, MA, USA) using a 5- to 12-MHz multifrequency linear-array transducer. After an acceptable image of near and far arterial walls were obtained, the transducer was secured and stabilized in a stereotatic clamp and small markings were made on the subject’s skin to ensure proper placement of the transducer for subsequent sessions. Ultrasound gain settings were not altered between measurements as changes during the session may have influenced image results [27]. The first arm measured immediately following exercise was randomly selected. The exercise sessions and FMD assessments were all performed in a dark, quiet, climate controlled (22-24° C) room.

Doppler brachial artery blood velocity was measured simultaneously with arterial diameters via ultrasound. Doppler flow signals were corrected at an insonation angle of 60° with the sample volume positioned in the middle of the artery. Diameter images and Doppler measurements for blood velocity were recorded for 60 seconds at baseline prior to cuff inflation. The automatic cuff was then rapidly inflated to 250 mm Hg and maintained for 5 minutes until cuff deflation. Diameter and blood velocity recordings recommenced prior to cuff Fdeflation and continued for 3 minutes after deflation. Ultrasound images were recorded at 5 frames/second using Camtasia (TechSmith, Okemos, MI, USA) and converted to an AVI file. The AVI files were also mirrored vertically using movie editing software (Windows Movie Maker, Microsoft Corporation, Redman, WA, USA) which allowed us to assess retrograde blood velocity.

### Brachial artery shear rate

Brachial artery SR was calculated using the following formula: 4 **·** Vm **·** D^-1^, where Vm is mean blood velocity (cm **·** s^-1^) and D is mean arterial diameter (cm) obtained via ultrasound. SR was assessed in both arms prior to exercise, at 5, 10, and 15 minutes into each exercise session, and for each FMD post-occlusion period. The mean of the 3 exercise SR measurements was used for analysis. The oscillatory shear index (OSI) was calculated for each SR assessment as follows: |retrograde SR|/(|retrograde SR| + |antegrade SR|) [28]. The shear rate area under the curve (SR_auc_) above baseline was calculated to quantify the hyperemic stimulus by summing the areas of successive post-occlusion trapezoids (with 3 s bases) until peak dilation for the post-occlusion phase of the FMD procedure [29].

### Data analysis

#### **Brachial artery diameters and blood velocities**

Automated edge-detection software (Brachial Analyzer, Medical Imaging Applications LLC, Coralville, IA, USA) was used to analyze off-line diameters and blood velocities as previously described [24]. This software allows the technician to determine a region of interest where the near and far vessel walls are clearest. The vessel wall borders are detected using an optimal graph search-based segmentation that uses a combination of pixel density and image gradient as an objective function. Analyzed images were reviewed by the technician and edited when needed to confirm that diameter measures were always determined from the intima-lumen interface at the near and far vessel wall. Blood velocities were determined by choosing a region of interest that encompassed the Doppler wave form and a trace of the wave form was automatically selected. Mean blood velocity was calculated for each cardiac cycle using the trace of the velocity-time integral. All measurements were performed by a single technician who was blinded to the treatment condition for each image file. Diameters and blood velocities were not EKG-gated [30]. FMD was calculated as the peak diameter following cuff deflation and was determined using the highest 3-sec moving average and presented as a percentage change from baseline diameter. The time to peak (TTP) dilation was determined from cuff deflation to peak diameter.

#### **Statistical analysis**

The sample size (n = 12) was based on pilot data from a comparable study design where we found FMD of 3.93 ± 1.1% following exercise with high forearm cuff pressure and 6.43 ± 2.2% after exercise with low cuff pressure. We made the assumption that oxidative stress accounted for 70% of the pilot data effect size (f = 1.2; based on the variance explained by the effect and within session variance) with a power of 0.80 and alpha level of 0.05. To investigate the effect of forearm cuff pressure on baseline, exercise, and post-exercise measures, 3 × 2 (exercise session × arm) repeated measures ANOVA were utilized. To investigate the effect of placebo and vitamin C supplementation on baseline, exercise, and post-exercise variables, 2 × 2 (exercise session × arm) repeated measures ANOVA were used. When a significant ANOVA interaction or main effect was found, the Fisher-Hayter post hoc procedure was used to correct for multiple comparisons. Partial eta squared (η^2^) was determined for ANOVA interactions and main effects as an estimate of effect size. Within-arm planned comparison paired *t*-tests were performed for pre versus post-exercise FMD measures as well as for pre versus exercise variables. All values were expressed as the mean ± standard error of the mean. The alpha level for statistical significance was set *a priori* at 0.05.

## Results

Subjects were 26.3 ± 0.9 years old, were normotensive (systolic blood pressure: 117.6 ± 3.0 mm Hg, diastolic blood pressure: 73.4 ± 3.2 mm Hg), and had a normal fasting glucose and lipid profile (serum glucose: 89.4 ± 1.5 mg/dL; total cholesterol: 159.9 ± 4.7 mg/dL; HDL: 54.1 ± 3.4 mg/dL; LDL: 88.0 ± 4.4 mg/dL; VLDL: 17.8 ± 2.5 mg/dL; triglycerides: 89.1 ± 12.4 mg/dL). Subjects were lean (body mass index 22.6 ± 0.7 kg/m^2^) and participated in aerobic exercise for 314 ± 42 minutes/week.

Brachial artery blood velocity data are presented in Table 1. Heart rate, brachial artery diameters, SR_auc_, and TTP data are presented in Table 2. 

**Table 1:** Retrograde, antegrade, and mean blood velocity at baseline and during exercise

	**Baseline**		**Exercise**	
	**Control**	**Cuffed**	**Control**	**Cuffed**
Retrograde BV (cm/s)				
20 mmHg	2.6 ± 0.51	2.6 ± 0.57	10.1 ± 1.10^	11.0 ± 1.34^*
40 mmHg	2.4 ± 0.42	2.3 ± 0.54	9.8 ± 0.98^	13.4 ± 1.20^*
60 mmHg	2.9 ± 0.47	2.8 ± 0.54	11.1 ± 1.01^	14.8 ± 1.22^†*
60 mmHg + Vit C	2.4 ± 0.42	2.2 ± 0.41	10.4 ± 0.78^	14.5 ± 1.17^*
Antegrade BV (cm/s)				
20 mmHg	11.3 ± 2.11	11.5 ± 1.63	21.6 ± 2.53^	21.5 ± 2.08^
40 mmHg	8.6 ± 0.80	10.7 ± 2.25	19.1 ± 1.51^	21.0 ± 1.35^
60 mmHg	9.8 ± 0.80^ǂ^	10.1 ± 0.84^ǂ^	19.8 ± 1.35^	21.4 ± 1.48^*
60 mmHg + Vit C	8.0 ± 0.40	8.6 ± 0.56	18.0 ± 1.03^	21.9 ± 1.65^*
Mean BV (cm/s)				
20 mmHg	8.7 ± 2.40	8.9 ± 1.92	11.6 ± 3.20^	10.5 ± 2.70*
40 mmHg	6.2 ± 1.01	8.4 ± 2.43	9.3 ± 2.04^	7.5 ± 1.54*
60 mmHg	7.0 ± 0.84	7.3 ± 1.01	8.8 ± 1.88	6.6 ± 1.77*
60 mmHg + Vit C	5.7 ± 0.54	6.4 ± 0.72	7.6 ± 1.12	7.4 ± 1.56

Values are means ± SE. BV = blood velocity. ^Significantly different versus baseline. †Significantly different versus exercise cuffed 20 mm Hg. *Significantly different versus control arm. ǂSignificantly different versus vitamin C trial.

**Table 2:** Heart rate, brachial artery diameters, post-occlusion shear rate area under the curve, and post-occlusion time-to-peak

	**Baseline**		**Exercise**		**Post-Exercise**	
**Variable**	**Control**	**Cuffed**	**Control**	**Cuffed**	**Control**	**Cuffed**
Heart rate (bpm)						
20 mmHg	47 ± 2	―	100 ± 3^†	―	60 ± 3^	―
40 mmHg	48 ± 2	―	101 ± 2^†	―	55 ± 3^	―
60 mmHg	50 ± 2	―	100 ± 2^†	―	57 ± 3^	―
60 mmHg + Vit C	48 ± 2	―	100 ± 2^†	―	55 ± 2^	―
Diameters (mm)						
20 mmHg	4.0 ± 0.09	4.2 ± 0.10	4.0 ± 0.08	4.0 ± 0.09	4.0 ± 0.07	4.1 ± 0.10*
40 mmHg	4.0 ± 0.07	4.1 ± 0.10	4.0 ± 0.08	4.1 ± 0.09	4.0 ± 0.10	4.2 ± 0.10*
60 mmHg	4.1 ± 0.10	4.1 ± 0.05	4.0 ± 0.09	4.1 ± 0.07	4.0 ± 0.09	4.2 ± 0.06*
60 mmHg + Vit C	4.0 ± 0.09	4.1 ± 0.08	4.0 ± 0.09	4.1 ± 0.09	4.0 ± 0.09	4.1 ± 0.10
SR_auc_ (a.u.)						
20 mmHg	47.5 ± 3.69	57.4 ± 6.29*	―	―	64.8 ± 8.43^	67.9 ± 9.58
40 mmHg	45.5 ± 4.65	48.3 ± 5.22*	―	―	57.2 ± 7.44^	56.7 ± 5.41^
60 mmHg	45.7 ± 3.70	45.6 ± 3.91*	―	―	72.6 ± 12.91^	60.9 ± 6.97^
60 mmHg + Vit C	41.7 ± 3.37	54.0 ± 6.23*	―	―	55.5 ± 6.14^	61.5 ± 7.01
TTP (s)						
20 mmHg	35.0 ± 2.13	38.5 ± 2.50	―	―	47.8 ± 5.40^	50.0 ± 5.83^
40 mmHg	35.3 ± 2.84	36.5 ± 2.63	―	―	41.0 ± 3.74	40.5 ± 2.92
60 mmHg	36.8 ± 2.31	34.5 ± 1.25	―	―	45.0 ± 4.55	40.5 ± 3.19
60 mmHg + Vit C	35.8 ± 2.26	35.8 ± 2.00	―	―	41.5 ± 3.91	40.8 ± 3.86

Values are means ± SE. SR_auc_ = shear rate area uncer the curve until peak dilation (arbitrary units). TTP = time-to-peak dilation. ^Significantly different versus baseline. *Significantly different versus control arm. †Significantly different versus post-exercise.

### The Effects of Cuff Pressure on Brachial Artery Retrograde SR

**Figure 1:**
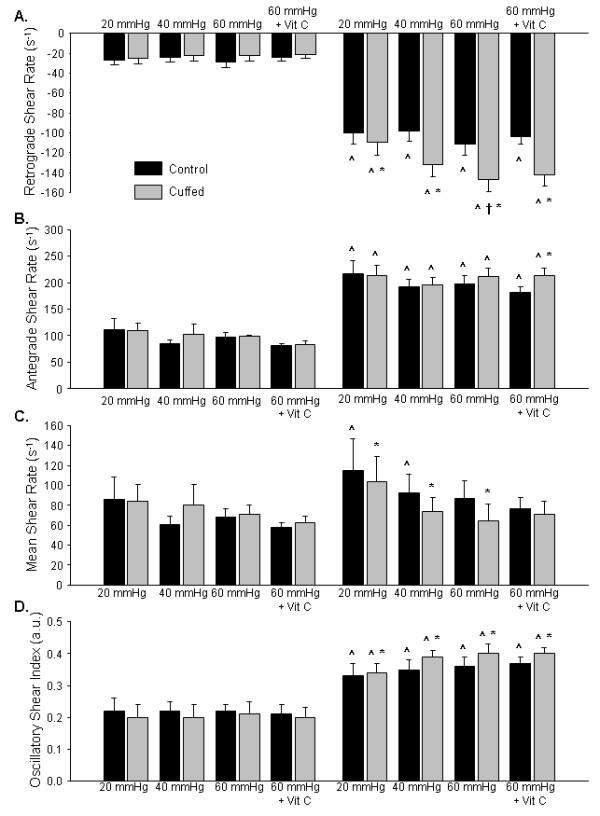
**(A) Retrograde shear rate, (B) antegrade shear rate, (C) mean shear rate, and (D) oscillatory shear index for both arms in the 20 mm Hg, 40 mm Hg, 60 mm Hg, and 60 mm Hg + vitamin C supplementation.** *Significantly different versus control arm. ^Significantly different versus baseline. †Significantly different versus 20 mmHg forearm cuffed arm.

Baseline and exercise brachial artery retrograde SR data are presented in Figure 1A. Baseline retrograde SR was not different across sessions or between arms (session × arm interaction: *P* = 0.97 (Greenhouse-Geisser correction), η^2^ < 0.01; session main effect: *P* = 0.60, η^2^ = 0.05; arm main effect: *P* = 0.55, η^2^ = 0.03). During the exercise sessions, the cuffed arm had greater retrograde SR versus the control arm and the 60 mm Hg cuff pressure session elicited a 33% greater retrograde SR in the cuffed arm versus the 20 mm Hg cuff pressure session (session × arm interaction: *P* = 0.10 (Greenhouse-Geisser correction), η^2^ = 0.21; session main effect: *P* = 0.02, η^2^ = 0.31; arm main effect: *P* < 0.01, η^2^ = 0.74).

### The Effects of Cuff Pressure on Brachial Artery Antegrade SR

Baseline and exercise brachial artery antegrade SR data are presented in Figure 1B. Antegrade SR was not different across sessions or between arms at baseline (session × arm interaction: *P* = 0.28, η^2^ = 0.11; session main effect: *P* = 0.50 (Greenhouse-Geisser correction), η^2^ = 0.05; arm main effect: *P* = 0.51, η^2^ = 0.04). During exercise, antegrade SR increased from baseline in both arms and all sessions (*P* < 0.01). Antegrade SR during exercise was not different across sessions or between arms (session × arm interaction: *P* = 0.23, η^2^ = 0.13; session main effect: *P* = 0.43 (Greenhouse-Geisser correction), η^2^ = 0.06; arm main effect: *P* = 0.52, η^2^ = 0.04).

### The Effects of Cuff Pressure on Brachial Artery Mean SR

Baseline and exercise brachial artery mean SR data are presented in Figure 1C. Mean SR was not different across sessions or between arms at baseline (session × arm interaction: *P* = 0.29, η^2^ = 0.11; session main effect: *P* = 0.61, η^2^ = 0.04; arm main effect: *P* = 0.41, η^2^ = 0.06). In the control arm, mean SR was 26% greater than baseline in the 20 mm Hg exercise session (*P* < 0.05) and 35% greater than baseline in the 40 mm Hg exercise session (*P* = 0.02). Mean SR during exercise was not different across sessions and the control arm mean SR was greater versus the cuffed arm (session × arm interaction: *P* = 0.75, η^2^ = 0.03; session main effect: *P* = 0.27 (Greenhouse-Geisser correction), η^2^ = 0.11; arm main effect: *P* = 0.02, η^2^ = 0.38).

### The Effects of Cuff Pressure on Brachial Artery OSI

Baseline and exercise brachial artery OSI data are presented in Figure 1D. At baseline, OSI was not different across sessions or between arms (session × arm interaction: *P* = 0.92, η^2^ < 0.01; session main effect: *P* = 0.95, η^2^ < 0.01; arm main effect: *P* = 0.24, η^2^ = 0.12). The OSI during exercise was greater than baseline for both arms and sessions (*P* < 0.01). During exercise, OSI was greater in the cuffed arm versus the control arm in each pressure condition, but there were no differences between sessions (session × arm interaction: *P* = 0.40, η^2^ = 0.08; session main effect: *P* = 0.20, η^2^ = 0.14; arm main effect: *P* = 0.01, η^2^ = 0.43).

### The Effects of Cuff Pressure on Brachial Artery FMD%

**Figure 2:**
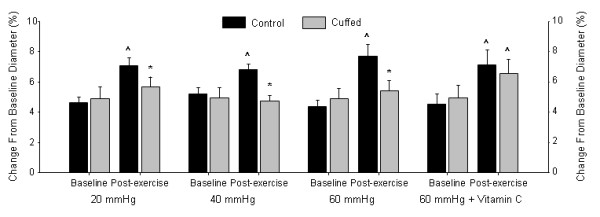
**FMD in both cuffed and control arms at baseline and post-exercise in the 20 mm Hg, 40 mm Hg, 60 mm Hg, and 60 mm Hg + vitamin C supplementation.** *Significantly different versus post-exercise control arm. ^Significantly different versus baseline.

Baseline and post-exercise brachial artery FMD data are illustrated in Figure 2. Baseline FMD was not different across exercise sessions or between arms (session × arm interaction: *P* = 0.72, η^2^ = 0.03; session main effect: *P* = 0.62, η^2^ = 0.04; arm main effect: *P* = 0.72, η^2^ = 0.01). Post-exercise FMD in the control arm was significantly greater than baseline for all exercise sessions (*P* < 0.01) and no significant increases from baseline were observed for the cuffed arm (*P* = 0.24). Post-exercise FMD was lower in the cuffed arm versus the control arm; however, FMD responses were not different across sessions (session × arm interaction: *P* = 0.43 (Greenhouse-Geisser correction), η^2^ = 0.06; session main effect: *P* = 0.41, η^2^ = 0.08; arm main effect: *P* < 0.01, η^2^ = 0.68).

### The Effects of Vitamin C on Brachial Artery Retrograde SR

Baseline and exercise brachial artery retrograde SR data are presented in Figure 1A. Retrograde SR in the 60 mm Hg conditions with and without vitamin C supplementation were not different across sessions or between arms at baseline (session × arm interaction: *P* = 0.82, η^2^ < 0.01; session main effect: *P* = 0.33, η^2^ = 0.09; arm main effect: *P* = 0.48, η^2^ = 0.05). Retrograde SR during exercise was greater than baseline in both arms and both sessions (*P* < 0.01). During the exercise sessions, the cuffed arm had greater retrograde SR versus the control arm, but the responses were not different between placebo and vitamin C sessions (session × arm interaction: *P* = 0.86, η^2^ < 0.01; session main effect: *P* = 0.32, η^2^ = 0.09; arm main effect: *P* < 0.01, η^2^ = 0.75).

### The Effects of Vitamin C on Brachial Artery Antegrade SR

Baseline and exercise brachial artery antegrade SR data are presented in Figure 1B. Antegrade SR in the 60 mm Hg conditions with and without vitamin C supplementation was not different between arms at baseline; however antegrade SR was greater in the placebo condition versus the vitamin C condition (session × arm interaction: *P* = 0.88, η^2^ < 0.01; session main effect: *P* = 0.02, η^2^ = 0.43; arm main effect: *P* = 0.80, η^2^ < 0.01). During exercise, antegrade SR was not different between the placebo and vitamin C sessions; however the cuffed arm antegrade SR was 18 % greater than the control arm (session × arm interaction: *P* = 0.11, η^2^ = 0.22; session main effect: *P* = 0.37, η^2^ = 0.08; arm main effect: *P* < 0.01, η^2^ = 0.48).

### The Effects of Vitamin C on Brachial Artery Mean SR

Baseline and exercise brachial artery mean SR data are presented in Figure 1C. Mean SR in the 60 mm Hg conditions with and without vitamin C supplementation was not different between sessions or between arms (session × arm interaction: *P* = 0.82, η^2^ < 0.01; session main effect: *P* = 0.16, η^2^ = 0.17; arm main effect: *P* = 0.65, η^2^ = 0.02). Mean SR during exercise was not different from baseline in both arms and both sessions (*P* > 0.09). During exercise, mean SR was not different between placebo and vitamin C sessions or between arms (session × arm interaction: *P* = 0.27, η^2^ = 0.11; session main effect: *P* = 0.81, η^2^ < 0.01; arm main effect: *P* = 0.11, η^2^ = 0.22).

### The Effects of Vitamin C on Brachial Artery OSI

Baseline and exercise brachial artery OSI data are presented in Figure 1D. At baseline, OSI in the 60 mm Hg conditions with and without vitamin C supplementation was not different between sessions or between arms (session × arm interaction: *P* = 0.88, η^2^ < 0.01; session main effect: *P* = 0.76, η^2^ = 0.01; arm main effect: *P* = 0.44, η^2^ = 0.06). The OSI during exercise was greater than baseline for both arms and sessions (*P* < 0.01). During exercise, OSI was 10 % greater in the cuffed arm versus the control arm, but there were no differences between sessions (session × arm interaction: *P* = 0.79, η^2^ < 0.01; session main effect: *P* = 0.88, η^2^ < 0.01; arm main effect: *P* = 0.02, η^2^ = 0.40).

### The Effects of Vitamin C on Brachial Artery FMD

Baseline and post-exercise brachial artery FMD data are illustrated in Figure 2. Baseline brachial artery FMD in the 60 mm Hg conditions with and without vitamin C supplementation were not different between the placebo and vitamin C sessions or between arms (session × arm interaction: *P* = 0.91, η^2^ < 0.01; session main effect: *P* = 0.81, η^2^ < 0.01; arm main effect: *P* = 0.52, η^2^ = 0.04). A significant session × arm interaction (*P* < 0.05, η^2^ = 0.32) for post-exercise FMD was observed. Post-exercise FMD was significantly greater in the control arm versus the cuffed arm following placebo treatment (*P* < 0.05); conversely there was no significant difference between the control and cuffed arm FMD following vitamin C supplementation (*P* > 0.05). Post-exercise FMD was unchanged versus baseline in the cuffed arm placebo condition (*P* > 0.05); whereas post-exercise FMD was elevated in control arm of both conditions (*P* < 0.05) and the cuffed arm in the vitamin C condition (*P* < 0.05).

## Discussion

The primary aims of this investigation were to (1) test the hypothesis that post-exercise brachial artery FMD would be attenuated in a dose-dependent manner by varying the dose of retrograde shear in the brachial artery during lower body exercise, and (2) test the hypothesis that vitamin C supplementation would prevent the attenuation of brachial artery FMD following elevated brachial artery oscillatory and retrograde shear during lower body exercise. The primary findings indicate that (1) brachial artery FMD is attenuated following exercise with oscillatory and retrograde shear above the normal response, but the attenuation is not different across the applied gradations of retrograde shear, and (2) vitamin C supplementation prevents the decline of FMD induced by augmented oscillatory and retrograde shear during exercise.

We did not observe a graded effect of cuff pressure on post-exercise FMD, despite achieving statistically different SR between the lowest and highest cuff pressures. In other words, the overall goal of applying different shear between exercise sessions was achieved but was not reflected in graded effects on post-exercise FMD. The lack of a dose effect on FMD is therefore interpretable as a true negative.

In a recent review, it was postulated that increased antegrade shear during exercise provides a beneficial stimulus to the endothelium which may counteract the possible negative influence of concurrent augmented retrograde shear [2]. The findings from the present study support this hypothesis. The greatest dose of retrograde shear did not further impair post-exercise FMD beyond what was observed for the lowest dose of retrograde shear. These results are in contrast to those obtained from augmented retrograde shear during rest and therefore refute our original hypothesis that FMD would be attenuated in a dose-dependent manner by various doses of retrograde shear in the brachial artery during lower body exercise. Thijssen et al. observed a dose–response relationship between brachial artery retrograde SR and brachial artery FMD at rest without a concurrent increase of antegrade shear [18]. Given these contrasting results, it appears that augmented antegrade shear associated with exercise partially ameliorates the deleterious effects of elevated oscillatory and retrograde shear on post-exercise endothelial function.

Augmented antegrade SR during exercise may reduce the impact of retrograde shear on post-exercise FMD by limiting the increase of the oscillatory pattern. During exercise in all conditions, the OSI was increased and the control arm was lower than the cuffed but there were no differences between cuff pressures. Therefore, the amount of oscillation during exercise appears to at least partially influence post-exercise FMD. This is supported by the lack of a difference of OSI between cuffed arms which resulted in post-exercise FMD that were equivalent. Tinken and colleagues performed two studies where brachial artery SR profiles were modified during handgrip exercise [7] and lower body cycling [23] using forearm compression. Although OSI was not calculated in either study, it appeared to increase in the cuffed arm in both investigations, primarily due to significantly lower antegrade shear as retrograde SR was unchanged during the interventions. These shear patterns resulted in post-exercise FMD that were either unaltered [7] or decreased from baseline [23]. In the present study, despite the increase in antegrade SR during exercise, slightly elevated OSI and retrograde SR during exercise attenuated post-exercise FMD. This indicates that even minimal increases in oscillatory and retrograde shear during exercise can substantially influence post-exercise endothelial function. Previous results [7,23] and those from the present study indicate that even minimal increases in retrograde SR and OSI above the normal response during exercise may play a prominent role restricting or reducing post-exercise FMD.

It is challenging to differentiate which component of the shear profile (i.e. antegrade, retrograde, mean, or OSI) during exercise provides the appropriate stimulus for improving post-exercise endothelial function. OSI, antegrade, and retrograde SR were elevated during all exercise conditions in the control arm which increased post-exercise FMD. Further augmentation of oscillatory and retrograde SR in the cuffed arm resulted in an attenuated post-exercise FMD. Tinken et al. [7] found an augmented post-exercise brachial artery FMD following handgrip exercise which increased antegrade, retrograde, mean shear, and presumably OSI. However, mean shear in the control arms of the present investigation did not increase during the 60 mm Hg sessions, yet post-exercise FMD was still augmented. Collectively, it appears as though exercise in these non-cuffed conditions elicited appropriate antegrade, retrograde and oscillatory shear responses which augmented post-exercise FMD.

The findings from this investigation demonstrate that vitamin C prevents an attenuated post-exercise FMD following elevated oscillatory and retrograde shear during exercise. The oscillatory and retrograde shear patterns induced by cuff pressure in the placebo condition likely produced reactive oxygen species which overcame local antioxidant defenses. Antioxidant status in the control arm appears to maintain or lower oxidative stress in response to the normal shear patterns induced by the exercise bout which improved FMD. Retrograde and oscillatory shear enhance reactive oxygen species production in cultured endothelial cells by increasing NADPH oxidase subunit mRNA expression and augmenting subunit phosphorylation [11,12], increasing production of reactive oxygen species by xanthine oxidase [31] and mitochondria [13] which leads to the oxidation of BH_4_ to BH_3_· radicals [14,32]. Low BH_4_ bioavailability uncouples eNOS thereby reducing eNOS function and augmenting eNOS superoxide production [32,33]. Vitamin C supplementation appears to preserve post-exercise NO bioavailability in response to augmented oscillatory and retrograde shear during exercise. Since vitamin C was only administered prior to the highest dose of retrograde shear we cannot explicitly state that oxidative stress contributes similarly to lower retrograde shear conditions. Beyond the antioxidant effects of vitamin C, it appears to reduce BH_3_· radicals back to BH_4_[34], which would improve eNOS production of NO [35].

### Limitations

Our study has several limitations. FMD data following exercise should be interpreted with caution due to several vascular alterations (i.e. basal arterial diameter, sympathetic tone, blood flow and shear stress, and reactive hyperemia) which potentially contribute to the results [36]. In particular, significantly greater post-exercise baseline diameters in the cuffed arm may have artificially confounded the FMD results due to the mathematical bias against large baseline diameters. However, we did assess FMD as the absolute change from baseline to post-occlusion peak diameter and found identical results (data not shown). Also, smooth muscle function was not assessed before or after exercise; however manipulated SR patterns during exercise using similar experimental procedures do not appear to alter smooth muscle function [7,23]. Notably, a circulating marker of oxidative stress was not measured to confirm a reduction in reactive oxygen species. Currently, there is no clear consensus for the optimal circulating biomarker of intracellular oxidative stress, and the relevance of measuring a circulating marker as an indication of the oxidative state within a cell may be questionable. Since we did not measure a marker of oxidative stress, we cannot clearly state that post-exercise FMD following vitamin C supplementation was preserved by a decreased oxidative stress. However, observations indicate that augmented oxidative stress is abolished when vitamin C is given prior to several different types of interventions [37-39]. Furthermore, post-exercise FMD in the cuffed arm with prior vitamin C supplementation was similar to the control arm in the placebo and vitamin C conditions, which indicates that NO bioavailability was unaffected. Finally, diet was not strictly controlled with our subjects; therefore dietary antioxidants may have varied between exercise sessions. To reduce this possible variation between sessions, our subjects fasted according to the latest dietary guidelines for FMD assessments [26] and were asked to maintain their normal dietary habits throughout the study.

## Conclusion

This study provides evidence that brachial artery flow-mediated dilation is attenuated following lower body exercise with oscillatory and retrograde shear above the normal response, but the attenuation is not different across the applied gradations of retrograde shear which were imposed. We interpret these findings to indicate that augmented antegrade shear in the brachial artery during lower body aerobic exercise can partially override the negative influence of retrograde shear on post-exercise flow-mediated dilation in the brachial artery by minimizing the increase of oscillatory shear. Furthermore, vitamin C supplementation prevented an attenuation of post-exercise flow-mediated dilation due to elevated retrograde shear during exercise. These data suggest that reactive oxygen species may contribute to the attenuation of brachial artery flow mediated dilation following lower body exercise with augmented brachial artery retrograde shear rate.

## Abbreviations

eNOS, Endothelial nitric oxide synthase; BH_4_, Tetrahydrobiopterin; FMD, Flow-mediated dilation; SR, Shear rate; OSI, Oscillatory shear index; TTP, Time to peak.

## Competing interests

The authors declare that they have no competing interests.

## Authors’ contributions

BDJ was responsible for conception and study design, data collection, data analysis, statistical analysis and interpretation, and drafting the article. KJM assisted with study design, statistical interpretation, and revised the article for intellectual content. SCN contributed to study design, statistical interpretation, and revised the article for intellectual content. TDM participated in study design, statistical interpretation, and revised the article for intellectual content. JPW contributed to the development of the scientific question, study design, statistical interpretation, and revised the article for intellectual content. All authors read and approved the final manuscript.
